# Artificial intelligence for MRI diagnosis of joints: a scoping review of the current state-of-the-art of deep learning-based approaches

**DOI:** 10.1007/s00256-021-03830-8

**Published:** 2021-09-01

**Authors:** Benjamin Fritz, Jan Fritz

**Affiliations:** 1 Department of Radiology, Balgrist University Hospital, Forchstrasse 340, CH-8008 Zurich, Switzerland; 2 Faculty of Medicine, University of Zurich, Zurich, Switzerland; 3 New York University Grossman School of Medicine, New York University, New York, NY 10016 USA

**Keywords:** Artificial intelligence, Deep learning, Neural networks, Computer, Magnetic resonance imaging, Musculoskeletal system, Joints

## Abstract

Deep learning-based MRI diagnosis of internal joint derangement is an emerging field of artificial intelligence, which offers many exciting possibilities for musculoskeletal radiology. A variety of investigational deep learning algorithms have been developed to detect anterior cruciate ligament tears, meniscus tears, and rotator cuff disorders. Additional deep learning-based MRI algorithms have been investigated to detect Achilles tendon tears, recurrence prediction of musculoskeletal neoplasms, and complex segmentation of nerves, bones, and muscles. Proof-of-concept studies suggest that deep learning algorithms may achieve similar diagnostic performances when compared to human readers in meta-analyses; however, musculoskeletal radiologists outperformed most deep learning algorithms in studies including a direct comparison. Earlier investigations and developments of deep learning algorithms focused on the binary classification of the presence or absence of an abnormality, whereas more advanced deep learning algorithms start to include features for characterization and severity grading. While many studies have focused on comparing deep learning algorithms against human readers, there is a paucity of data on the performance differences of radiologists interpreting musculoskeletal MRI studies without and with artificial intelligence support. Similarly, studies demonstrating the generalizability and clinical applicability of deep learning algorithms using realistic clinical settings with workflow-integrated deep learning algorithms are sparse. Contingent upon future studies showing the clinical utility of deep learning algorithms, artificial intelligence may eventually translate into clinical practice to assist detection and characterization of various conditions on musculoskeletal MRI exams.

## Introduction

In the field of artificial intelligence (AI), different classes of computer algorithms have been applied to carry out a broad variety of diagnostic radiology tasks. Artificial intelligence’s seemingly limitless possibilities have generated enormous interest among radiologists and imaging scientists with a constantly increasing number of annual publications [1].

Specifically, AI algorithms that learn from data without human intervention offer exciting prospects for musculoskeletal radiology, including improvements in productivity, diagnostic performance, health preservation, disease prediction, and imaging utilization.

Within AI, deep learning (DL) algorithms can learn many different tasks that directly apply to musculoskeletal radiology, including image reconstruction, synthetic image creation, tissue segmentation, and detection and characterization of musculoskeletal diseases and conditions on radiographs, ultrasonography, CT, and MR images.

The interest in DL algorithms for disease detection and image interpretation is primarily based on two value propositions: improving the diagnostic performance of image interpretations by reducing the 3–5% human error rate and expediting image interpretation and report generation [2].

Although musculoskeletal MRI interpretations of subspecialized radiologists have high accuracies [3, 4], many years of training are usually required to attain proficiency for the broad range of musculoskeletal MRI exams. Disease-detecting DL algorithms may aid in providing expert-level interpretations for readers with less expertise and may also play a role in teaching residents and fellow [5].

However, within musculoskeletal radiology, the largest number of disease-detecting DL algorithms has been applied to interpreting radiographs, whereas a comparably smaller number of studies have been published on DL algorithms interpreting musculoskeletal MRI exams.

The lower number of studies and regulatory agency-approved DL algorithms for musculoskeletal MRI exams is likely based on the substantially higher complexity, variability, and number of images of MRI exams when compared to radiography.

There is an almost infinite variety of MRI protocols for each joint based on local preference, pulse sequence technology, and available equipment. Many pulse sequence parameters substantially influence signal, contrast, and anatomical detail of musculoskeletal MR images, including field strength, repetition time, echo time, matrix size, slice thickness, field-of-view, acceleration, coil technology, flip angle, echo train lengths, bandwidths, and vendor-specific image processing techniques [6, 7].

Notwithstanding the formidable challenges MRI poses for disease-detecting DL algorithms, several groups have developed pioneering DL algorithms to detect internal derangement on musculoskeletal MRI exams. The novel capabilities of this new category of DL algorithms have been demonstrated in several proof-of-concept studies, of which many may be on the verge of demonstrating proof-of-generalizability and proof-of-clinical-applicability.

We provide a clinically focused review of the current state of DL-based MRI diagnosis of joints.

## Knee MRI

The majority of DL algorithms for detecting and characterizing internal derangement on MRI have been developed for the knee joint, which is likely motivated by the high number of knee MRI exams, overall good image quality, high clinical impact, finite complexity of anatomy, standardized positioning, and defined set of common injuries.

From a clinical perspective, MRI of the knee yields high diagnostic accuracy for many osseous and soft tissue lesions, including radiographically occult fractures, ligament and meniscus tears, articular cartilage defects, neoplastic diseases, and synovial conditions, thus obviating the need for diagnostic arthroscopy in many cases.

### ACL tears

ACL tears are among the most frequent acute knee injuries. In the USA, the estimated incidence is 200,000 cases annually [8]. The diagnosis is primarily based on skilled clinical testing for knee instability. The clinical Lachman test is 81% sensitive and 81% specific for diagnosing a complete rupture of the ACL [9]. In addition to confirming an ACL tear, the main contributions of MRI is to characterize the tear type for surgical decision-making and diagnose concomitant knee injuries.

In 2017, one of the first studies demonstrated the ability of two machine-learning models to diagnose and differentiate lower-grade partial-thickness and full-thickness ACL tears [10]. The algorithms used preselected and annotated sagittal MR images that contained regions outlining the ACL. One algorithm achieved an area under the receiver operating characteristic curve (AUC) of 89% for diagnosing partial-thickness tears and 94% for full-thickness tears. This semi-automated approach demonstrated the ability to train a machine-learning algorithm for diagnosing and characterizing ACL tears on MRI with potentially clinically useful diagnostic accuracy. Several fully automated studies followed with increasingly promising results of refined algorithms.

In 2018, a study demonstrated the ability of a deep convolutional neural network (CNN) to diagnose a variety of knee injuries, including ACL tears [11]. The network used sagittal T2-weighted, coronal T1-weighted, and axial PD-weighted MR images and achieved a sensitivity of 76%, a specificity of 97%, and an AUC of 97% for diagnosing ACL tears. Radiologists had a significantly higher sensitivity of 91% and similar specificity of 93%. Notably, the study also evaluated the performance of radiologists working with the support of the DL algorithm (DL-augmented radiologists) and found that the DL algorithm significantly increased radiologists’ sensitivity by 5%, whereas the specificity remained unchanged. The DL algorithm was also applied to the publicly available external knee MRI data set “KneeMRI” [10]. The DL performance decreased to an AUC of 82% but could be increased to 91% after dedicated retraining with the external data set, highlighting the important role of external data sets to test the generalizability of DL algorithms.

A study published in 2019 presented a different CNN for fully automated ACL tear detection [3]. Using sagittal fat-suppressed proton density-weighted and sagittal fat-suppressed T2-weighted 3-Tesla (T) MR images, the model achieved a sensitivity of 96%, a specificity of 96%, and an AUC of 98%. In comparison, the participating radiologists achieved sensitivities of 96–98% and specificities of 96–98%, whereas a resident achieved a lower specificity of 90%. There was no significant performance difference between the CNN and radiologists.

Another study published in the same year evaluated different CNNs for diagnosing full-thickness ACL tear on coronal proton density-weighted MR images without fat suppression [12]. Cases with mucoid degeneration and partial-thickness ACL tear were excluded. Ground truth was established by a musculoskeletal radiologist. The best performance was achieved when cropping the MR images to the ACL region rather than using full-size MR images. The model using five contiguous MR images performed best, achieving a sensitivity of 100% and specificity of 93% in a small test set. Comparisons to human readers were not reported.

In 2020, a study described a customized CNN for diagnosing ACL tears using sagittal fat-suppressed proton density-weighted 1.5-T and 3-T MR images [13]. The CNN achieved a sensitivity of 98%, specificity of 94%, and accuracy of 96%. On the same data set, senior radiologists achieved a sensitivity of 96% and a specificity of 86%, but testing for statistically significant differences was not reported.

**Fig. 1:**
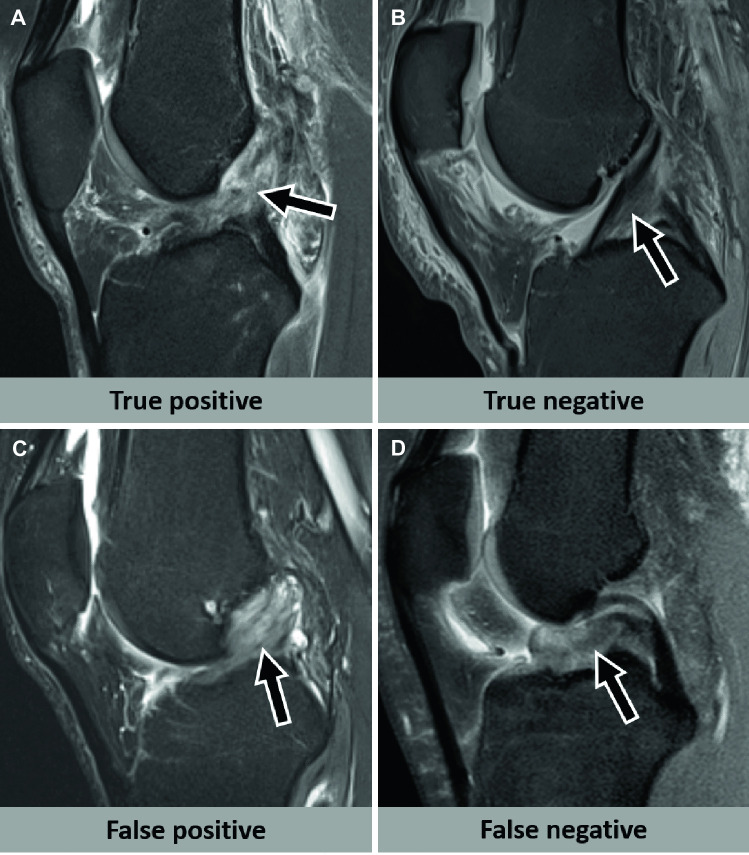
Deep learning algorithm evaluation of the anterior cruciate ligament. **A** A 46-year-old woman who sustained an acute knee injury during tennis. Sagittal fat-suppressed proton density-weighted MR image shows an arthroscopy-confirmed full-thickness anterior cruciate ligament tear (arrow), which was correctly diagnosed by the deep learning algorithm (true positive). **B** A 40-year-old man who sustained an acute knee injury during ice hockey. Sagittal fat-suppressed proton density-weighted MR image shows an arthroscopy-confirmed intact anterior cruciate ligament (arrow), which was correctly diagnosed by the deep learning algorithm (true negative). **C** A 40-year-old man with chronic knee pain. Sagittal fat-suppressed proton density-weighted MR image shows mucoid degeneration of an intact anterior cruciate ligament (arrow), which was erroneously diagnosed as a tear by the deep learning algorithm (false positive). **D** A 19-year-old man with acute knee pain after fall. Sagittal fat-suppressed proton density-weighted MR image shows a full-thickness anterior cruciate ligament tear with a displaced bucket-handle tear of the lateral meniscus, resembling a double posterior cruciate ligament sign. The displaced meniscus fragment in the intercondylar notch (arrow) may be the underlying reason for the CNN assessing the anterior cruciate ligament erroneously as intact (false negative). Data were derived with a deep learning algorithm described in a study published by Germann et al. [4]

In the same year, a study described a serial CNN for detecting ACL tears in a fully automated fashion, using coronal and sagittal fat-suppressed fluid-sensitive MR images and arthroscopic surgery as the reference standard [4] (Fig. 1). Using a more homogenous in-house test data set of 1.5-T and 3.0-T MRI exams, the CNN achieved a sensitivity of 99%, specificity of 94%, and AUC of 97%. The diagnostic performance of the CNN was lower for a heterogeneous external test data set consisting of 234 outside knee MRI exams from over 50 different institutions, achieving a sensitivity of 93%, specificity of 87%, and AUC of 90%. Three musculoskeletal radiologists had significantly higher diagnostic performances with sensitivities of 97–98%, specificities of 99–100%, and AUCs of 98–99% for either test set.

Until that point in time, most CNNs used two-category classifications of the ACL into intact versus tear. In 2020, a multi-class CNN described a hierarchical severity staging of four different ACL patterns, including intact, partial-thickness tear, full-thickness tear, and ACL graft following reconstruction. The CNN achieved a sensitivity of 97–100% and a specificity of 100% for identifying ACL grafts. For intact ACL, the CNN sensitivities were 89–93% and specificities were 88–90%, whereas the CNN achieved sensitivities of 76–82% and specificities of 94–100% for full-thickness ACL tears [14]. The ground truth was based on radiological assessments, and comparisons to human readers were not reported.

Several additional studies using variations of publicly available and custom architecture CNNs reported sensitivities and specificities between 85 and 95% [15–17]. Table 1 summarizes key characteristics and performance levels of a group of representative studies.

**Table 1:** Summary of AI studies for fully automated anterior cruciate ligament tear detection

					Diagnostic performance of AI algorithm			Diagnostic performance of human readers			
Study	Reference standard	Labels	Analyzed pulse sequences	Field strengths [T]	Sensitivity	Specificity	AUC	Sensitivity	Specificity	AUC	Comments
Bien et al. [11]	Radiologist interpretation	Intact, tear	Sag T2, cor T1, ax PD	1.5, 3.0	76%	97%	96%	91%	93%		AUC of DL algorithm with an external validation set (“KneeMRI”) was 0.82, which increased to 0.91 after retraining Radiologists had significantly higher sensitivity, whereas specificities were similar
Chang et al. [12]	Radiologist interpretation	Intact, tear	Cor PD	1.5, 3.0	100%	93%					Study excluded mucoid degeneration and partial-thickness tear
Liu et al. et al. [3]	Surgical inspection	Intact, tear	Sag PD, sag fat-suppressed T2	3.0	96%	96%	98%	96–96%	98%	98%	No significant differences of performance metrics between AI and radiologists
Germann et al. [4]	Surgical inspection	Intact, tear	Sag and cor fat-suppressed fluid-sensitive	1.5, 3.0	96%	93%	94%	98%	100%	90%	Radiologists had significantly higher specificity, whereas sensitivities were similar
Irmakci et al., 2020 [17]	Radiologist interpretation	Intact, tear	Sag T2, cor T1, ax PD	1.5, 3.0	78%	94%	95%				
Zhang et al. [13]	Surgical inspection	Intact, tear	Sag fat-suppressed PD	1.5, 3.0	98%	94%	96%	96%	86%		Statistical differences between DL algorithm and radiologists were not reported
Namiri et al. [14]	Radiologist interpretation	Intact, partial tear, full-thickness tear, graft	3D fat-suppressed PD	3.0	76–82%	94–100%					Numbers refer to full-thickness tear versus any other category
Tsai et al. [15]	Radiologist interpretation	Intact, tear	ax PD	1.5, 3.0	92%	89%	96%				
Awan et al. [16]	Radiologist interpretation	Intact, partial-thickness tear, full-thickness tear	Sag fat-suppressed PD	1.5	92%	95%	98%				

The best performance is shown in the table for studies with multiple diagnostic performance metrics and different models. For studies that included human readers with differing experience levels, the diagnostic performances of the most experienced readers are shown in the table

*Sag* sagittal, *cor* coronal, *ax* axial, *PD* proton density, *DL* deep learning, *AUC* area under the receiver operating curve, *3D* three-dimensional, *AI* artificial intelligence

**Fig. 2:**
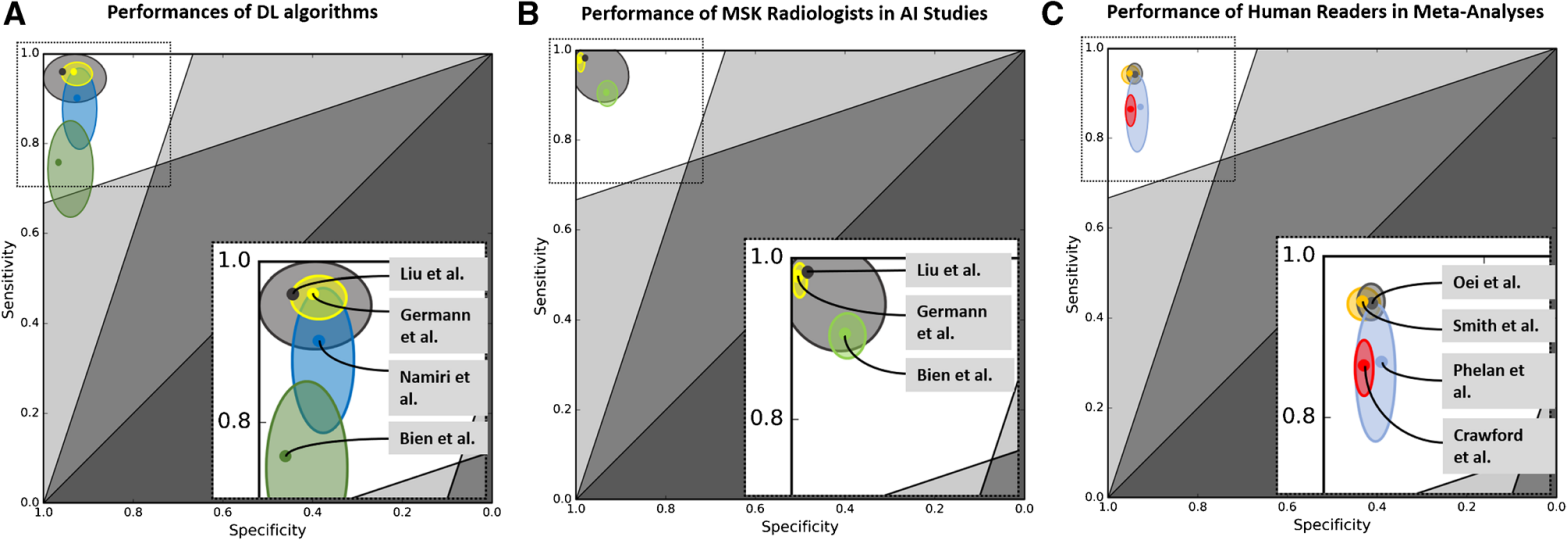
Comparative performances of AI and human readers for MRI diagnosis of anterior cruciate ligament tears. Plots show the diagnostic performances of deep learning (DL) algorithms (**A**), musculoskeletal radiologists participating in artificial intelligence (AI) studies (**B**), and meta-analyses of human readers (**C**) for anterior cruciate ligament tear detection. The solid dots indicate the estimates of sensitivities (y-axis) and specificities (x-axis). The surrounding ellipses represent the corresponding 95% confidence intervals. Most studies are located exclusively in the left upper zone (white background), indicating at least acceptable diagnostic performance for diagnosis [62]. Right lower cut-out boxes represent a magnification of the left upper area (dashed box). In **A** and **B**: dark gray = Liu et al. [3]; yellow = Germann et al. [4]; blue = Namiri et al. [14]; green = Bien et al. [11]. In **C**: gray = Oei et al. [18]; orange = Smith et al. [20]; light blue = Phelan et al. [21]; red = Crawford et al. [19]. Note: Only studies reporting 95% CI were included. Test data set rules, settings, reference standards, and experience levels of readers differed between studies, which may limit the direct comparability of diagnostic performances

Four meta-analyses from 2003 to 2016 estimating the diagnostic performances of human readers for detecting ACL tears derived pooled sensitivities of 87–95% and specificities of 93–95% [18–21] (Fig. 2). Comparing human readers with proof-of-concept studies of DL algorithms may suggest similar performances for diagnosing ACL tears (Fig. 2). However, this assumption requires caution, as there is a paucity of studies evaluating the generalizability and clinical applicability of DL algorithms.

Studies attempting to evaluate real-life scenarios by including heterogeneous and external MRI test sets of varying field strengths, pulse sequence protocols, and vendors found decreasing diagnostic performances, suggesting that diagnostic performances of deep learning algorithms may not easily translate and be lower in clinical practice [4, 11].

The performance level of fellowship-trained musculoskeletal radiologists is likely substantially higher than meta-analyses using historical data and heterogeneous reader pools suggest [3, 4] (Fig. 2). The clinical usefulness of DL algorithms will likely scale with the experience level of readers, where readers with less expertise benefit more and musculoskeletal radiologists with advanced expertise benefit less [5, 11].

In addition, most published studies compared the diagnostic performance of DL algorithms *against* radiologists, whereas the more like practice scenario will be radiologists working *with* DL algorithms [11].

To understand potential efficiency gains afforded by DL-based ACL diagnosis, more studies using realistic study designs emulating the daily work of radiologists with implementation into their practice environment will be needed.

### Meniscus tears

Like ACL injuries, meniscus tears are among the most common indications for knee MRI [22]. Meniscus tears may be due to acute trauma, but most are degenerative [23]. The prevalence of degenerative meniscus tears is 19% in the 6th decade of life and increases to 56% in individuals between the age of 70 and 90 [23]. While meniscus tears can be pain generators and incite inflammation and dysfunction, some meniscus tears, such as coapted horizontal tears, are often asymptomatic [24–26]. In the USA, approximately 50% of arthroscopic knee surgeries are performed to treat meniscus tears, indicating high socioeconomic importance [27].

Physical examination tests diagnose meniscus tears with 60–70% sensitivity and 70–71% specificity [28]. MRI can confirm a clinically suspected meniscus tear in indeterminate cases and identify additional or alternative abnormalities. However, potentially more important contributions of MRI include the characterization of the tear depth, location, pattern, tissue quality, length, and the amount and integrity of previous partial meniscectomy or meniscal repair.

Several studies describe the ability of deep learning algorithms for binary meniscus classification into tear and no tear.

In 2018, a CNN was described to detect meniscus tears using axial, sagittal, and coronal stacks of two-dimensional fast spin echo MR images of different contrast weightings [11]. The tear laterality was not part of the reporting. Radiological interpretation served as the standard of reference. The CNN achieved a sensitivity of 71%, a specificity of 74%, and an AUC of 85%. In comparison, participating radiologists and orthopedic surgeons performed significantly better with a sensitivity of 82%, a specificity of 88%, and an accuracy of 85%.

Two studies published in 2019 as part of a meniscus tear detection challenge of the French Radiology Society describe deep learning algorithms to detect meniscus tears. Both studies used the same test data set consisting of single preselected and annotated sagittal fat-suppressed T2-weighted MR images [29, 30], achieving AUCs of 91% and 94%, respectively, for diagnosing the presence of a meniscus tear and characterization of the tear orientation into vertical or horizontal.

Also in 2019, the feasibility of fully automated CNN-based meniscus tear detection on sagittal PD-weighted three-dimensional MR image reformations was demonstrated [31]. Since only sagittal reformations were analyzed, only meniscus lesions of the anterior and posterior horns were included. The laterality was not reported. With radiologists serving as the reference standard, the CNN achieved a sensitivity of 82%, specificity of 90%, and AUC of 89% for detecting a meniscus tear. The CNN also applied a three-class model for severity staging, which achieved an accuracy of 82% for intact menisci, 78% for mild-to-moderate tears, and 75% for severe tears.

**Fig. 3:**
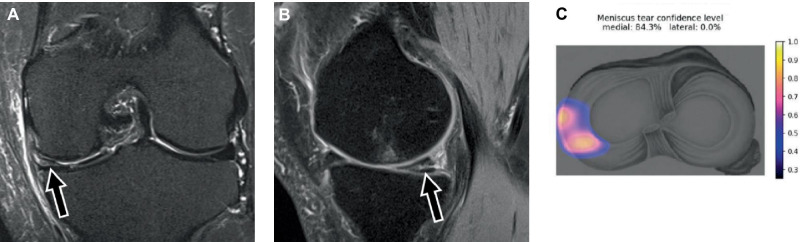
MRI of the left knee joint in a 59-year-old patient with chronic medial knee pain. **A** Coronal short tau inversion recovery (STIR) MR image through the mid-body segment of the medial meniscus shows a horizontal cleavage tear (arrow). **B** Sagittal proton density-weighted MR image with spectral fat suppression shows the horizontal meniscus tear extending to the posterior horn of the medial meniscus (arrow). **C** AI-based assessment of the medial and lateral menisci predicted a medial meniscus tear with a probability of 84%. The heat map located the tear (colored area) correctly to the mid-body segment and junction to the posterior horn (**C**). Arthroscopic knee surgery confirmed the meniscus tear. Data were derived with a deep learning algorithm described in a study published by Fritz et al. [32]

In 2020, a fully automated CNN was clinically evaluated to detect and differentiate medial and lateral meniscus tears on coronal and sagittal fat-suppressed fluid-sensitive MR images [32]. In contrast to other published studies evaluating AI for meniscus tear detection, arthroscopic surgery was used as the reference standard. The CNN achieved a sensitivity of 84%, a specificity of 88%, and an AUC of 78% for the medial meniscus (Fig. 3). For lateral meniscus tears, the CNN achieved a sensitivity of 58%, a specificity of 92%, and an AUC of 78%. Compared to musculoskeletal radiologists, the specificities for medial and lateral meniscus tears were similar; however, the sensitivities of the musculoskeletal radiologists were approximately 10% higher in each compartment.

In 2021, a different CNN was described for detecting meniscus tears on coronal and sagittal fat-suppressed PD-weighted MR images [33]. Radiologists served as the standard of reference. On an internal test data set, the CNN achieved sensitivities of 89%, specificities of 84%, and AUCs of 93% for detecting medial meniscus tears, and 67%, 88%, and 84% for detecting lateral meniscus tears, respectively. Using the previously published external test data set “MRNet” [11], the overall performance decreased to a sensitivity of 77%, specificity of 84%, and an AUC of 83% without differentiating medial from lateral meniscus tears. Retraining with the external data set improved the performance to 81%, 87%, and 89%, respectively. As an additional feature, the CNN was trained to detect displaced meniscus fragments, achieving a sensitivity of 80% and specificity of 85% for the medial meniscus, and a sensitivity of 57% and specificity of 95% for the lateral meniscus.

Two other studies evaluated DL algorithms for meniscus tear detection using the publicly available “MRNet” data set [11]. For meniscus tear detection without differentiating laterality, one study using various CNNs achieved sensitivities of 62–69% and specificities of 76–81% [17]. The other study’s CNN achieved a sensitivity of 86% and specificity of 89% by reportedly using coronal T1-weighted MR images only, which, however, are least accurate for radiologists to diagnose meniscal tears [15].

Based on meta-analyses, human readers achieve pooled sensitivities of 89–93% and specificities of 81–88% for diagnosing medial meniscus tears and 76–79% and 93–96% for lateral meniscus tears, respectively [18, 19, 21].

**Fig. 4:**
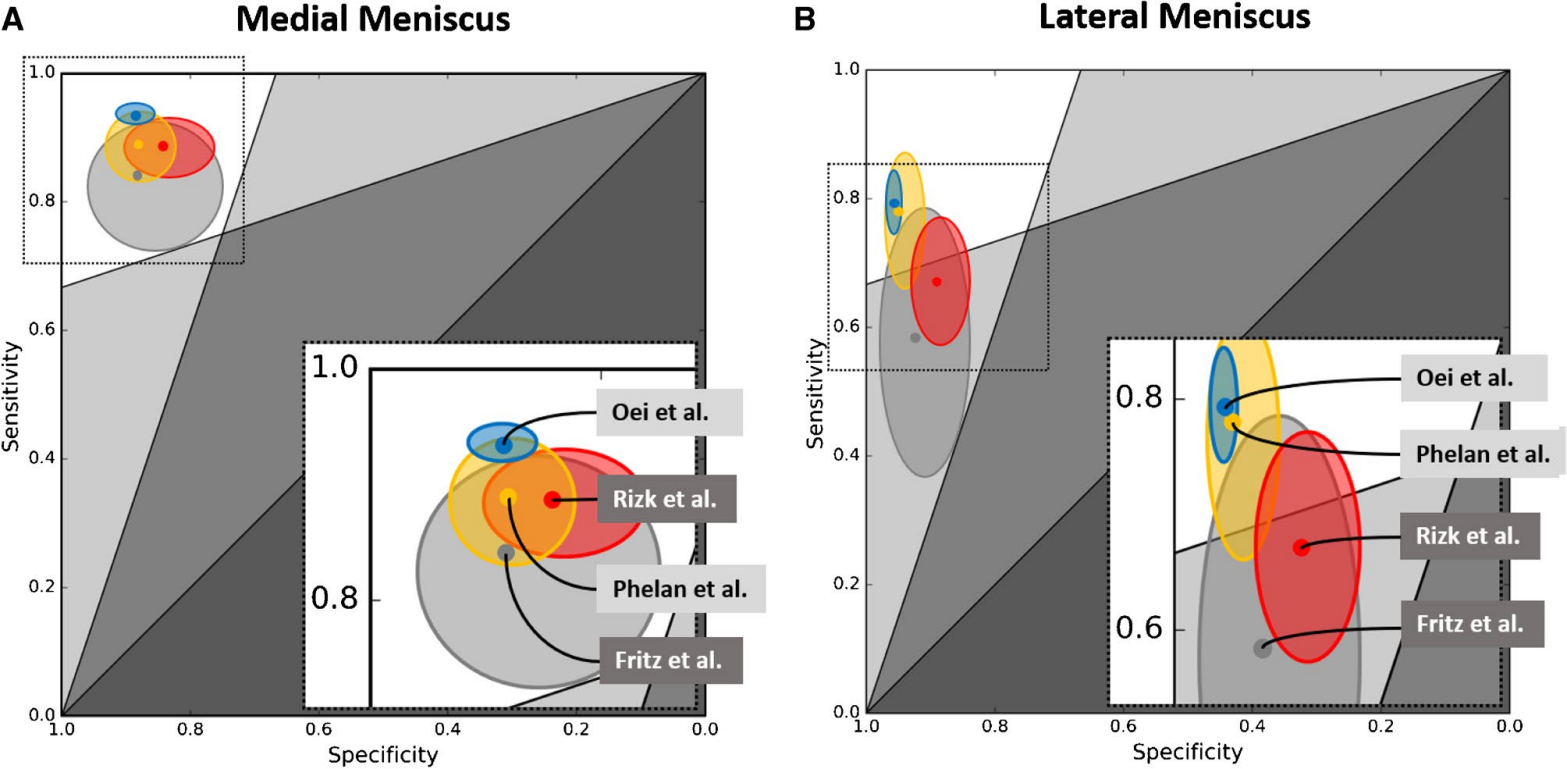
Comparative performances of AI (red and gray circles) and human readers (yellow and blue circles) for MRI diagnosis of meniscus tears. Plots show the diagnostic performances of selected deep learning (DL) algorithms and meta-analyses of human readers for MRI-based diagnosis of medial (**A**) and lateral (**B**) meniscus tears. The solid dots indicate the estimates of sensitivities (y-axis) and specificities (x-axis). The surrounding ellipses represent the corresponding 95% confidence intervals. For medial meniscus tears (**A**), all studies are located exclusively in the left upper zone (white background), indicating at least acceptable diagnostic performance for diagnosis [62]. For lateral meniscus tears, the performance estimates of the two DL studies of Fritz et al. and Rizk et al. occupy the left lower zone (light gray background), indicating a limited sensitivity for clinical application. Right lower cut-out boxes represent a magnification of the left upper area (dashed box). In **A** and **B**: gray = DL algorithm of Fritz et al. [32]; red = DL algorithm of Rizk et al. [33]; yellow = meta-analysis of Phelan et al. [21]; blue = meta-analysis of Oei et al. [18]. Note: Only studies reporting 95% CI and differentiating between the medial and lateral meniscus were included. Test data set rules, settings, reference standards, and experience levels of readers differed between studies, which may limit the direct comparability of diagnostic performances

Published data suggest that CNNs may achieve similar diagnostic performance parameters than human readers to diagnose medial and lateral meniscus tears (Table 2) (Fig. 4). However, similar to CNN-based detection of ACL tears, various factors require consideration.

**Table 2:** Summary of AI studies for fully automated meniscus tear detection

						Diagnostic performance of AI algorithm			Diagnostic performance of human readers			
Study	Reference standard	Label	Analyzed sequence	Field strengths [T]		Both	Med	Lat	Both	Med	Lat	Comments
Bien et al. [11]	Radiologist interpretation	Intact, tear	Sag T2, cor T1, ax PD	1.5, 3.0	Sensitivity Specificity AUC	71% 74% 85%	-	-	82% 88% -	-	-	
Pedoia et al. [31]	Radiologist interpretation	Intact, tear	3D fat-suppressed PD	3.0	Sensitivity Specificity AUC	82% 90% 89%	-	-	-	-	-	Severity grading into no tear, mild-moderate tear, and severe tear achieved accuracies of 81%, 78%, and 75%, respectively
Fritz et al. [32]	Surgical inspection	Intact, tear	Sag and cor fat-suppressed fluid-sensitive	1.5, 3.0	Sensitivity Specificity AUC	91% 87% 96%	84% 88% 88%	58% 92% 78%	94% 90% 92%	95% 88% 92%	69% 97% 83%	Performances of human readers are given as averages
Rizk et al. [33]	Radiologist interpretation	Intact, tear	Cor fat-suppressed PD, sag fat-suppressed PD	1.0, 1.5, 3.0	Sensitivity Specificity AUC	-	89% 84% 93%	67% 88% 84%	-	-	-	Validation with external “MRNet” data set [11] achieved a sensitivity, specificity, and AUC of 77%, 84%, and 0.83, respectively
Irmakci et al. [17]	Radiologist interpretation	Intact, tear	Sag T2, cor T1, ax PD	1.5, 3.0	Sensitivity Specificity AUC	62–69% 76–81% 78–81%	-	-	-	-	-	
Tsai et al. [15]	Radiologist interpretation	Intact, tear	Cor T1	1.5, 3.0	Sensitivity Specificity AUC	86% 89% 90%	-	-	-	-	-	

*Sag* sagittal, *cor* coronal, *ax* axial, *PD* proton density, *TSE* turbo spin echo, *AUC* area under the receiver operating curve, *3D* three-dimensional, *AI* artificial intelligence, *both* both menisci combined, *med* medial meniscus, *lat* lateral meniscus

To estimate the diagnostic performance of deep learning algorithms in clinical practice, the use of an independent standard of reference may be critically important. There is a paucity of studies using a surgical reference standard [32], whereas most studies reference radiological interpretation. While this is a practical approach that compares diagnostic performance levels to radiologists, the clinical standard of reference for MRI interpretations is surgical inspection.

The use of clinically realistic test data sets, including heterogeneous MRI exams acquired with different field strength, variable MRI protocols, and different pulse sequence techniques, is another important factor. Similar to trends with ACL tears, the application of DL algorithms to external data sets will likely result in lower diagnostic performances than found with internal test data sets [33].

While assigning the laterality to meniscus tears is a fundamental requirement, the MRI characterization of meniscus tears, including location, type, and the degree of tissue degeneration, are equally important features for future AI-based algorithms to add clinical value.

### Other abnormalities

Few studies have reported the diagnostic performance of CNNs for diagnosing other internal derangement abnormalities. One study reported a sensitivity of 88% and specificity of 71% for detecting osteoarthritis, joint effusions, iliotibial band syndromes, bone contusions and fractures, posterior cruciate ligament tears, the presence of a plica, and medial collateral ligament tears without differences in performance compared to general radiologists [11].

Using the same “MRNet” data set but different CNN, two other studies describe different performances for detecting the same knee abnormalities, including a higher sensitivity of 97% and similar specificity of 72% [15], as well as a higher sensitivity of 97% but markedly lower specificities of 28–40% [17].

## Shoulder MRI

After the knee, the shoulder is the second most common site of joint pain and the second most common referral for joint MRI in many departments worldwide [34].

Many conditions can be diagnosed and successfully managed with history, skilled clinical examination, and radiography evaluation. However, MRI and MR arthrography are the most accurate tests for non-invasive internal derangement assessment in indeterminate and recalcitrant cases, including the rotator cuff, long head biceps tendon, glenoid labrum, glenohumeral ligaments, capsule, and articular cartilage [35]

In addition to the presence of a rotator cuff tear, differentiation of bursal surface from articular surface partial-thickness tears, partial-thickness from full-thickness tears, tear size, degree of tendon fiber retraction, and grade of muscle bulk atrophy and fatty infiltration are important MRI characteristics that aid in surgical decision-making and prognosis.

A growing number of studies describe the use of deep learning algorithms for diagnosing rotator cuff tears and segmenting rotator cuff muscles.

### Rotator cuff tear detection

Rotator cuff tear incidence increases with age and is present in over 70% of patients with shoulder pain over the age of 70 [36]. Small rotator cuff tears may heal with conservative treatments, whereas more advanced rotator cuff tears may require surgical repair.

MRI contributes information on several cuff-related factors that influence the success of surgical cuff repair to relieve pain and retain or restore shoulder mobility, including tear size (anteroposterior tear length, mediolateral tear length, and tear size area), tear depth, tendon quality, tendon fiber retraction, fatty infiltration of the rotator cuff muscles, and the number of torn tendons [37, 38]. For example, tear size is associated with surgical repair success rate, which can vary from 79% for small to 24% for massive rotator cuff tears [39, 40].

A network meta-analysis of 144 diagnostic studies determining the diagnostic performances of MRI and MR arthrography for diagnosing rotator cuff tears estimated pooled sensitivities of 80–87% and specificities of 81–90% [41]. MRI and MR arthrography had higher performances than ultrasonography.

In 2019, a CNN was described for rotator cuff detection. The network was trained on almost 2000 patients, used a three-dimensional approach based on native coronal two-dimensional T2-weighted MR images, and differentiated normal tendon, partial-thickness tears, and full-thickness tears [42]. Radiologists served as the standard of reference. Using a homogenous data set from a single institution, the three-dimensional deep learning approach achieved a high diagnostic accuracy of 87% and an AUC of 96%, outperforming several baseline machine-learning approaches. There was no comparison with the diagnostic performance of radiologists.

In 2020, another CNN also using a three-dimensional approach was described for automated rotator cuff tear detection on axial T1-weighted and sagittal and coronal fat-suppressed T2-weighted MR images [43]. The CNN employed a 2-class categorization into intact and torn tendons and a 5-class categorization into intact tendons, partial-thickness tears, and small-size, medium-size, and large-size full-thickness tears. The CNN was trained on 1924 exams and tested on a data set of 200 exams. Surgical inspection served as the standard of reference. For 2-class categorization, the CNN achieved a sensitivity of 94%, a specificity of 90%, and an accuracy of 93%. In comparison, surgeons achieved sensitivities of 86–90%, significantly lower specificities of 29–58%, and significantly lower accuracies of 68–76%. For 5-class categorization, the CNN achieved a sensitivity of 92% and a specificity of 86%, whereas the surgeons achieved sensitivities of 89–93% and significantly lower specificities of 26–61%. Surgeons required between 20 and 34 s for interpretation, whereas the algorithm required a fraction of a second. The diagnostic performance of the CNN was compared with surgeons but not with radiologists.

### Rotator cuff muscle segmentation

The preoperative degree of atrophy and fatty infiltration of the rotator cuff muscles are predictors that are inversely associated with the long-term functional outcome of rotator cuff repair [44]. Furthermore, the progression of rotator cuff muscle atrophy and fatty infiltration after surgical repair correlates with poor functional outcomes [45]. As the commonly used Goutallier classification agreements vary between studies and measurements are time-consuming to obtain [46–49], automated deep learning-based quantification could add clinical value through improved reproducibility and efficiency gains.

In 2019, a study described a fully convolutional deep learning algorithm for segmenting the boundaries of the supraspinatus fossa and the supraspinatus muscle bulk [50]. Using sagittal T1-weighted MR images, the algorithm allowed for calculating the supraspinatus occupation ratio as a surrogate marker for supraspinatus muscle atrophy [51]. The algorithm achieved 99.8% pixel-wise accuracies and high similarity with manual segmentation (Dice coefficients of 0.94–0.97), promising automation, and improved efficiency for this task.

In 2020, a study described fully automated deep learning segmentation of supraspinatus, infraspinatus, subscapularis, and deltoid muscle on three-dimensional T1-weighted gradient-echo MR images in pediatric patients with neuromuscular diseases. Compared with manual segmentation, the algorithm achieved Dice similarity scores of 82%, 82%, 71%, and 83% for deltoid, infraspinatus, supraspinatus, and subscapularis muscles, respectively [52].

**Fig. 5:**
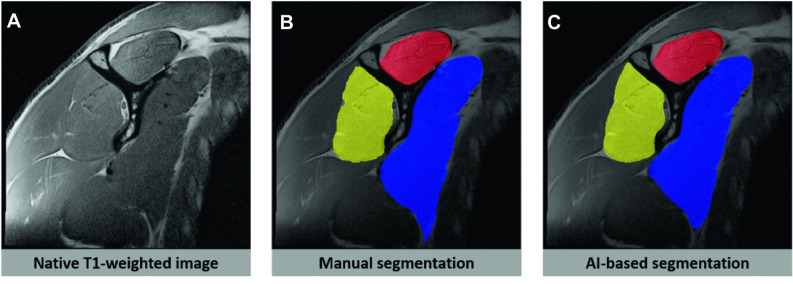
Sagittal T1-weighted MR image of the right shoulder (**A**), with manual (**B**) and AI-based (**C**) segmentations of the subscapularis (blue overlay), supraspinatus (red overlay), and infraspinatus/teres minor (yellow overlay) muscles. The manual (**B**) and AI-based (**C**) segmentations had high similarity with a Dice score > 0.93. The segmentation was derived with an AI-based algorithm described by Medina et al. [49]. Images courtesy of Martin Torriani M.D., Harvard Medical School, Massachusetts General Hospital, Boston, MA

In a 2021 study, two serial deep learning algorithms successfully identify the most suitable sagittal T1-weighted MR images resembling Y-views and subsequently segmented the subscapularis, supraspinatus, and infraspinatus/teres minor muscles (Fig. 5) [49]. The fully automated algorithms performed the tasks with greater than 98% accuracy to select an appropriate Y-view, and there was a high similarity with human manual segmentation on internal (Dice score greater than 0.96) and external (Dice score greater than 0.93) data sets, which build the foundation for future AI-based MRI quantification of muscle atrophy and classification of fatty infiltration.

## Other joints and musculoskeletal MRI applications

There is a paucity of published deep learning applications for joints other than the knee and shoulder. However, deep learning solutions for disease detection and quantification may similarly add value for higher volume MRI exams of the ankle, foot, and hip.

### Ankle

In 2019, a deep learning approach was described for detecting tears and monitoring healing of the Achilles tendon on MRI [53]. MRI exams of 30 healthy and 60 participants with Achilles tendon rupture were used for training, validation, and testing. In addition, postoperative MRI exams were included. The best network had a sensitivity, specificity, and accuracy greater than 97%, respectively, for binary classification of intact and torn tendons. In addition, the ability for MRI assessment of tendon healing following surgical repair was shown.

### MR neurography

In 2019, deep learning-based, fully automated segmentation of the sciatic nerve was demonstrated on MR neurography exams using axial non-fat-suppressed T2-weighted images of the thigh [54]. The CNN was trained on 42 participants with sciatic neuropathy and ten healthy participants, demonstrating human-level segmentation accuracy in 1 s, whereas human segmentation required 19 min. Automated segmentation of nerves could be an important step for quantitative MR neurography, which may improve objectivity in MR neurography-based diagnosis of sciatic neuropathy and monitoring of treatments.

Similarly, deep learning-based segmentation has been described for thigh muscles and wrist bones [55, 56].

### Oncology

MRI is accurate for detecting and defining the extent of musculoskeletal neoplasms but has lower accuracies for typing and grading neoplasms, predicting treatment response, and detecting tumor recurrence.

Different computer-aided detection and radiomics techniques have been applied over the past 5 years with mixed success [57–59]. Probabilistic artificial intelligence algorithms have been used successfully to determine bone tumors with the use of external data, such as reference of anatomic and demographic statistics [60]. Applying DL-based approaches may improve the diagnostic performance of MRI for those tasks.

In 2019, a deep learning algorithm was described for predicting local recurrence of giant cell tumor of bone after curettage based on pre-surgical MRI [61]. The model was trained on pre-surgical T1-weighted and T2-weighted MR images of 56 participants, which were followed for 6 years. The algorithm had an accuracy of 76% to predict tumor recurrence, which increased to 79% with age and location inclusion using a regression model. Four radiologists had a mean accuracy of 64%. In this study, data augmentation was used to improve training with a small data set, which might be a key technique to train algorithms with small available data sets inherent to rare diseases.

## Clinical perspective

Deep learning-based analysis of joint MRI exams is an emerging field of artificial intelligence. Several studies describe promising techniques to master the high complexity of MRI data, many of which primarily focus on the binary classification of the presence or absence of a feature. However, in many clinical situations, the added value of MRI for surgical decision-making and outcomes is often based on the characterization of an abnormality, such as tear location, configuration, size, and quality of a torn ligament, tendon, or meniscus. As such, algorithms with multi-class features, such as differentiating normal, partial-thickness, and small, moderate, and large full-thickness tears, may prove most useful in clinical practice.

Regardless of the features, an important question is who will benefit from algorithms for DL-based MRI diagnoses? The study-based diagnostic performance analyses of current deep learning algorithms for ACL and meniscus tear detection approach may not exceed the diagnostic performances of radiologists. While studies demonstrate high diagnostic performances of musculoskeletal radiologists, the added value of disease-detecting DL algorithms may scale with the radiologist’s expertise using it, which is an interesting topic for future studies.

Many research studies naturally report the initial performance of DL algorithms based on homogenized data sets, which are dissimilar from the daily mix of MRI exams in clinical practice and thus may not translate.

The use of a surgical standard of reference may be equally important for results to translate into clinical practice.

Many studies evaluated the performance of DL algorithms compared to radiologists, rather than comparing the performance of radiologists without and with using DL algorithms, which is the more likely practice scenario.

Studies emulating real-life practice settings, including readers with different levels of expertise and surgical reference standards, are needed to understand better the added value of DL-based MRI diagnosis.

Efficiency gains may be highest for DL algorithms performing tasks that require human operators a long time, such as segmentation of muscles and nerves. The speed of DL-based segmentation and quantification of muscle volume and fatty atrophy may eventually facilitate including this information routinely in radiology reports.

Additional efficiency gains may be delivered through AI-based pre-population of radiology reports, as dictating reports may require radiologists more time than interpreting MR images.

The combination of radiomics features and artificial intelligence may improve the performance and prediction power of MRI in evaluating musculoskeletal neoplasms, which could move MRI closer to being a tool for virtual tissue biopsy.

## Practice integration

Practice integration, including interfacing with scanner technology, hospital and radiology information systems, and picture archiving and communication system (PACS), and compatibility with departmental workflows, may be equally important for the clinical utility of the DL algorithms than diagnostic performance and efficiency gains.

**Fig. 6:**
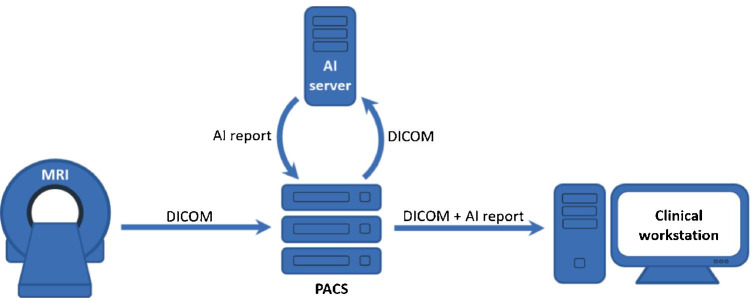
Artificial intelligence (AI) workflow as deployed at our institution. After acquiring the MRI study, the digital imaging and communications in medicine (DICOM) images are sent to the picture archiving and communication system (PACS). From the PACS, DICOM images can be routed to the local AI server either manually or based on the fulfillment of predefined criteria, such as DICOM header descriptions. After processing, the AI server sends the report as a PDF document back to the PACS

**Fig. 7:**
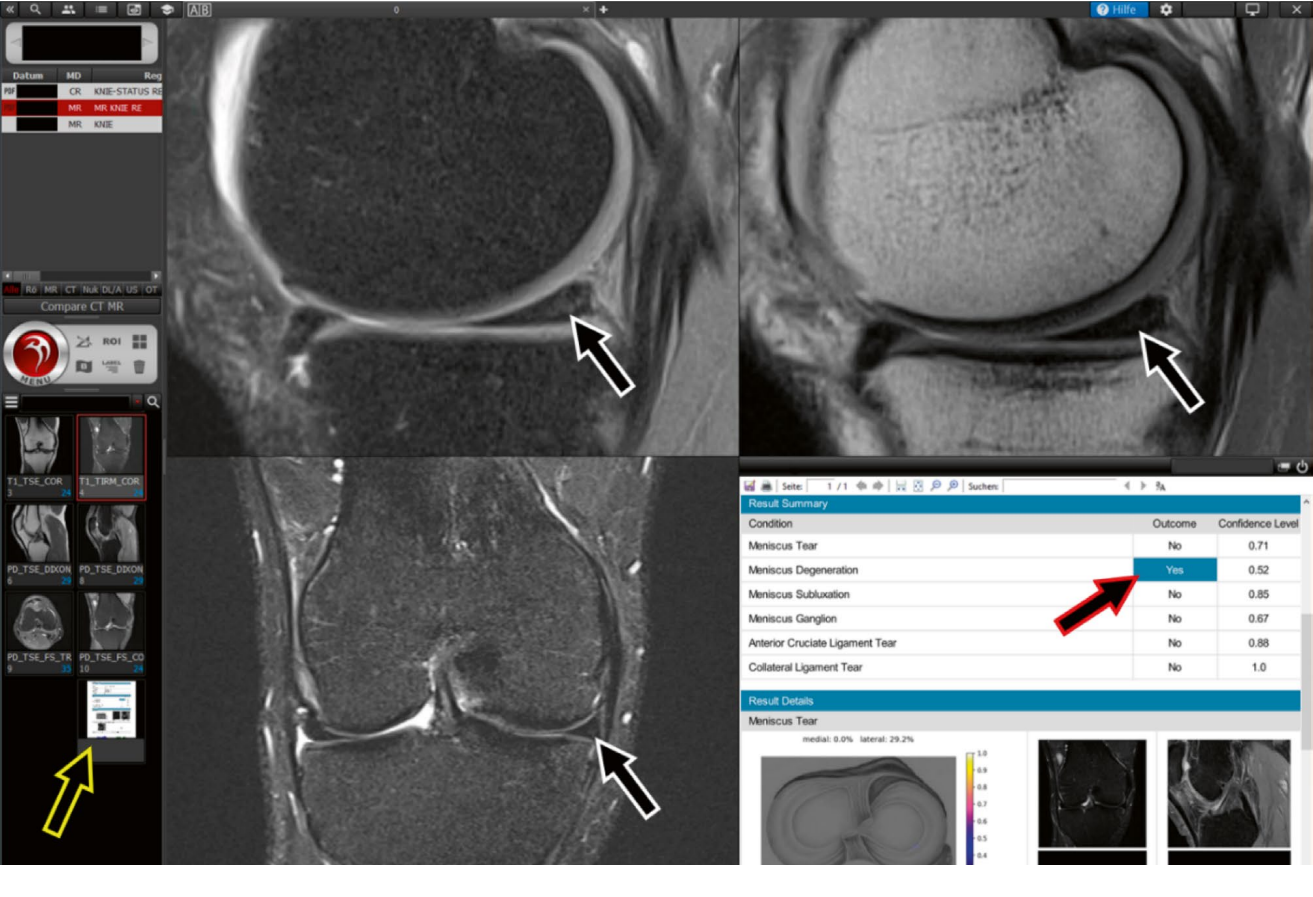
Artificial intelligence (AI)-augmented knee MRI interpretation using an investigational AI algorithm. The AI report appears as part of the MRI study in the left column (yellow-framed arrow) and can be displayed in a viewport (lower right viewport) or separate window (not shown). In this patient, the AI algorithm predicted internal degeneration of the medial meniscus with a probability of 52% (red-framed arrow), based on the intrasubstance signal hyperintensities (white-framed arrows), as well as absent meniscus tear, meniscus extrusion (subluxation), meniscus ganglions cyst, anterior cruciate ligament tear, and medial collateral ligament tear

Figure 6 resembles the current structure in our institution for the evaluation of DL algorithms for MRI diagnoses. All MRI studies can automatically be routed to the AI server and DL algorithm, or select studies may be routed manually by the radiologist. While routing every MRI exam may create high network traffic and work the DL server to full capacity, manual routing may create dead time and longer turnaround times than without using DL algorithms. Result reports may be added in various formats to the MRI study (Fig. 7).

## Picture this…

…at some point in the future, there may be DL algorithms for MRI diagnosis of many different anatomical structures, injury patterns, and pathological conditions fully integrated into departmental workflows. DL algorithms will fully prepopulate MRI report templates using predefined syntax and terminology upon opening MRI studies. Abnormal findings will be highlighted and linked with the respective images, which are already annotated with arrows and quantifying measurements. Binary DL algorithms for detecting the presence or absence of an abnormality will have evolved to include detailed characterizations and quantifications of abnormalities and additionally incorporate ancillary patient-specific and big data for determining urgency, predicting associated injuries, supporting treatment decisions, and rendering prognosis. At this stage, DL algorithms will have truly changed the practice of musculoskeletal MRI interpretation.

Our predictions for 5 years from now: There will be an increasing number of DL algorithms for MRI diagnoses of internal derangement approved for use in clinical practice, focusing mostly on detecting major abnormalities with limited complexity. DL algorithms will focus on large-volume MRI studies, such as the knee and shoulder, whereas a paucity of DL algorithms for MRI studies with smaller volumes and more complex anatomy will exist. There will be increasing but incomplete knowledge of which readers benefit from DL algorithms and what applications translate efficiency gains into clinical practice. Practice integration, maintenance and support, and search for viable business models to bear the cost will remain obstacles for broad use of DL algorithms.

Our predictions for 10 years from now: The number of DL algorithms for MRI diagnoses of internal derangement has increased detecting a broad variety of internal derangement of large joints, and a growing number of DL algorithms for diagnosing internal derangement of smaller joints, with improved abilities for pre-population of radiology reports. Improving integration options supported by most PACS, RIS, and HIS vendors result in more institutions using DL algorithms for MRI diagnoses, which eventually results in lower cost. Based on an improved understanding of the usefulness of AI, institutions will implement DL algorithms based on local expertise and practice patterns. Radiologists may slowly change their practice pattern from primary detection of abnormalities to supervision and quality control of DL-based detection.

## Conclusion

Deep learning-based analysis of joint MRI exams is an emerging field of artificial intelligence, which offers many exciting possibilities for musculoskeletal radiology. Current DL algorithms for MRI diagnoses of internal derangement focus on the detection of ACL tears, meniscus tears, and rotator cuff tears, as well as rotator cuff muscle segmentation. Additional studies are needed to understand the added value of deep learning-based MRI diagnoses in clinical practice, including reader expertise and teaching of residents and fellow.
